# An exploratory study of embitterment in traumatized refugees

**DOI:** 10.1186/s40359-021-00599-2

**Published:** 2021-06-10

**Authors:** Julia Spaaij, Matthis Schick, Richard A. Bryant, Ulrich Schnyder, Hansjörg Znoj, Angela Nickerson, Naser Morina

**Affiliations:** 1 Department of Consultation-Liaison-Psychiatry and Psychosomatic Medicine, University Hospital Zurich (USZ), University of Zurich (UZH), Culmannstrasse 8, 8091 Zurich, Switzerland; 2 School of Psychology, UNSW Australia, Sydney, NSW Australia; 3 University of Zurich, Zurich, Switzerland; 4 Department of Psychology, University of Bern, Bern, Switzerland

**Keywords:** Post-migration living difficulties, Moral injury, Embitterment, Self-efficacy, Refugees, Asylum seekers

## Abstract

**Background:**

Refugees and asylum seekers are frequently exposed to violence, human rights violations and unstable living conditions before, during, and after their displacement. Elevated prevalence rates of psychiatric disorders in forcibly displaced persons are well documented. However, less is known about other problems related to common refugee experiences, such as embitterment, moral injury, and diminished self-efficacy, and how they are related to trauma exposure and post-migration living difficulties.

**Methods:**

A cross-sectional sample of 71 refugees and asylum seekers in treatment were examined regarding exposure to potentially traumatic events, post-migration living difficulties, moral injury appraisals, self-efficacy, and embitterment.

**Results:**

Elevated levels of embitterment were reported by 68% of participants. The regression analysis revealed that greater moral injury appraisals and low levels of self-efficacy were significantly associated with higher levels of embitterment.

**Conclusion:**

The results provide first insights into embitterment and associated factors in refugee populations. Furthermore, they highlight the significance of moral transgressions and low levels of self-efficacy emerging from displacement and traumatic experiences for the development of mental health problems in a clinical sample of refugees. The findings have implications for future research, policy development and clinical practice.

**Supplementary Information:**

The online version contains supplementary material available at 10.1186/s40359-021-00599-2.

## Background

The number of armed conflicts has peaked since 2014, causing around 76,000 registered fatalities in 2018 alone [1]. In consequence, the amount of people forcibly displaced by violence and adversity has reached a record high of 80 million, amongst them more than 30 million refugees and asylum seekers [2]. By definition, refugees are exposed to persecution and, in consequence, to potentially traumatic experiences (PTE), such as imprisonment, torture and other forms of human rights violations [3, 4]. A dose–response relationship between trauma exposure and mental health problems is well-established [5–7]. Accordingly, refugees show high prevalence rates of trauma-related disorders, such as posttraumatic stress disorder (PTSD), depression and anxiety [8].

Even after arriving in presumably safe host countries, refugees and asylum seekers are confronted with ongoing stressors, such as insecure visa status, worries about family members back home or financial struggles, often summarily referred to as post-migration living difficulties (PMLD) [9–12]. Many studies have shown that PMLD contribute to the development and persistence of mental health problems over and above trauma exposure [10, 13–16]. Moreover, the high prevalence of mental disorders in forcibly displaced populations seems to persist even years after resettlement [15], suggesting that needs regarding treatment and support are not sufficiently met [17, 18].

While trauma exposure, PMLD, and their relation to posttraumatic stress, depression, and anxiety disorders, have received consistent scientific attention, far less is known about other constituents of refugee mental health and their potential as treatment targets. Preliminary evidence shows that, among others, moral injury, loss of control, self-efficacy, pain, attachment problems, and prolonged grief are associated with psychological impairment in refugees [19–26]. Despite this evidence, the interrelation of these aspects and their therapeutic value is poorly understood.

One clinically relevant consequence of the typical refugee experience of injustice, loss of control, and insecurity, is embitterment. Embitterment is referred to as a strong feeling of injustice and disappointment, combined with the urge to defend oneself, but with the inability to do so [27, 28]. Embittered individuals feel that they have been wronged and try to seek vengeance, yet they can feel cornered and helpless [28, 29]. According to Znoj [30], individuals who are prone to embitterment, experience their situation as hopeless and feel that their locus of control is external, resulting in the perception that they are unable to change their current state. The feeling of uncontrollability is frequently observed in the refugee context, as victims of human rights violations (e.g. torture) are likely to show alterations in their perception of control, which might then be reflected, amongst other things, in low levels of perceived self-efficacy [31]. The concept of self-efficacy refers to the perceived ability to achieve desired goals and successfully deal with adversities in life and has been found to play an important role in posttraumatic recovery [32, 33].

Even though embitterment is a feeling which is known to almost everyone [34], scientific research and clinical recognition of embitterment are scarce. In 2003, Linden and colleagues have described a condition called “posttraumatic embitterment disorder” (PTED) [28] which differentiates between transient and clinically relevant feelings of embitterment. Even though it has been discussed whether (posttraumatic) embitterment should be included into common diagnostic systems of mental health disorders [28, 35–38], it can only be coded among “Reaction to severe stress, and adjustment disorders” so far [27, 39].

According to Linden and colleagues, clinically relevant embitterment is present in 2.5–3% of non-clinical samples in Germany [40]. In clinical samples, the prevalence rate of embitterment is much higher, climbing to over 40% [41]. Studies on the relationship between embitterment and psychopathology have found that embittered individuals also show higher prevalence rates of other mental health problems [42].

A matter of great interest is to identify why some individuals exposed to injustice, hopelessness, and uncontrollability show resilience, whereas others are stuck and develop chronic feelings of embitterment. As embitterment seems to be associated with powerlessness and loss of control [30], one potential mechanism leading to high levels of embitterment could be a diminished sense of self-efficacy. Furthermore, according to Linden and colleagues [28, 43], perceived injustice seems to be the main trigger of embitterment. Therefore, they suggest that the mechanism turning the transient feeling of embitterment into a chronic mental health condition might be the violation of basic moral principles and beliefs [28, 43]. The violation of basic moral principles and core beliefs is in the literature referred to as “moral injury” and has been widely discussed in research with veterans [44]. However, the concept of appraising a negative event as a moral transgression has recently been applied to the refugee context as well [19, 26, 45, 46].

In view of the growing global number of refugees and the high occurrence and persistence of psychiatric disorders in these particularly vulnerable populations, it is of utmost importance to better understand the mental health consequences and the underlying psychological mechanisms emerging from the typical refugee experience. Due to their experiences before, during and after displacement, refugees might be at an increased risk to suffer from high, clinically relevant embitterment. A study by Linden and Teherani [47] comparing embitterment levels in Iranian political refugees and Iranian emigrants supports this hypothesis. Therefore, the aim of this explorative study was to assess embitterment and its associations with trauma exposure, post-migration living difficulties, as well as moral injury appraisals and general self-efficacy in a clinical sample of refugees and asylum seekers in Switzerland.

## Methods

### Participants

The sample consisted of 71 refugees and asylum seekers from various countries of origin. The participants were completers of a follow-up study (sample size at T1: *N* = 134). At T2, 44 individuals of the original sample could not be contacted anymore, and 19 individuals refused to further participate in the follow-up study (for details of the study flow see [48]). The data presented in this paper were collected as part of a larger longitudinal study on emotion regulation in traumatized refugees (see [10, 19, 23, 48]). Only cross-sectional data from T2 were examined in this study [48]. The participants were in treatment at the psychiatric outpatient units for victims of torture and war in Zurich and Bern, Switzerland, and received trauma-focused as well as non-trauma-focused psychotherapeutic interventions. Some were additionally treated with medication. The participants were ≥ 18 years old and needed to speak one of the study languages (English, German, Turkish, Arabic, Farsi and Tamil). Individuals who suffered from current psychosis, severe dissociative symptoms or acute suicidality were excluded from the study. The exclusion criteria were assessed before participation during a clinical interview by a trained clinical psychologist or psychiatrist. The data was collected in 2015 and 2016.

### Measures

The measures were presented in English, German, Turkish, Arabic, Farsi or Tamil. To meet high-standard translation procedures, the measures were translated and blind back-translated by professional translators [49]. Inconsistencies were addressed by the research team and independent bilingual individuals who had experience in working with health-related questionnaires.

#### Trauma exposure

Exposure to potentially traumatic events (PTE) was assessed by combining items from two standardized measures: The Harvard Trauma Questionnaire [50] and the first part of the Posttraumatic Diagnostic Scale [51]. The 23 types of traumatic events relevant to the refugee context, such as “*torture*” or *“Murder of a friend or family member”* were rated on a scale from 1 to 4 (1 = *“experienced myself”*; 2 = *“witnessed”*, 3 = *“heard of”*; 4 = *“neither nor”*). Only items rated as *“experienced myself”* were considered as exposure to trauma and were summed up to an overall count of PTE exposure.

#### Post-migration living difficulties

Post-migration living difficulties were assessed with the Post-Migration Living Difficulties Checklist [12, 52]. The checklist has been adapted to the Swiss context and consisted of 17 items [10]. Participants specified whether various post-migration challenges had been a problem during the past year. The items were rated on a 5-point Likert scale (1 = *“was not a problem / did not happen”* to 5 = *“very serious problem”*). To calculate an overall count of living difficulties, all items rated at least 3 (*“moderately serious problem”*) were considered as a problem.

#### General self-efficacy

The General Self-Efficacy Scale [53] was used to assess levels of self-efficacy. The 10 items were rated on a 4-point Likert scale (1 = *“not at all true”* to 4 = *“exactly true”*) and summed up to an overall sum score, with a higher score indicating higher levels of general self-efficacy. The measure has been validated cross-culturally [54] and has been used in the refugee context before [55]. The internal consistency was α = 0.95 in the present sample.

#### Moral injury appraisals

The Moral Injury Appraisals Scale is an adapted version of the Moral Injury Events Scale [56], which has been used with refugees and asylum seekers [19, 45]. The measure encompasses two subscales: The Moral Injury—Other subscale (5 items) and the Moral Injury—Self subscale (6 items). The Moral Injury—Other subscale measures whether the individual experienced events performed by others which transgressed his or her personal moral beliefs whereas the Moral Injury—Self subscale identifies actions performed by the individuals themselves which violated their core concepts. Only the Moral Injury—Other subscale was included in the current analysis. Each item was rated on a scale from 0 to 3 (0 = *“not at all”* to 3 = *“very much”*). The items were summed up with higher scores indicating greater moral injury—other appraisals. Cronbach’s alpha for the Moral Injury—Other subscale was 0.95 in the present sample.

#### Embitterment

The questionnaire used in the study was developed as a short version of the Bern Embitterment Inventory [57] by Znoj and Schnyder [58]. The original scale consists of 18 items and four subscales. More information on the validation procedures of the BEI can be found in Znoj [30]. The items selected for the short version of the BEI were highly correlated with the original scale and at the same time showed high internal consistency (α = 0.84). The short version consists of six items measured on a 5-point scale (0 = *“not true at all”* to 4 = *“extremely true”*). In the manual of the Bern Embitterment Inventory, the developers suggest a preliminary cut-off value of a mean of 2.2 which differentiates between elevated, clinically relevant and non-pathological embitterment [57]. For the development of the short version of the questionnaire, the correlation between the mean value of the short version and the mean value of the original scale was calculated and proved to be very high (*r* = 0.91). Thus, for this study we assumed that the cut-off value for elevated embitterment can also be applied to the short version of the BEI. Neither the original scale of the BEI [30] nor the short version of the BEI [58] have been used with refugee populations so far. Therefore, we ran a factor analysis to test the assumption that all six items load on one factor. A total sum score was calculated to represent embitterment symptom severity. The internal consistency was α = 0.84 in the present sample.

### Procedures

The study received ethics approval from the Ethics Committees of the Cantons of Zurich (Project Nr. KEK-ZH-Nr. 2011–0495) and Bern (Project Nr. KEK-BE_Nr. 152/12). Study team members and therapists approached potential participants who fulfilled the inclusion criteria and invited them to participate. All participants provided written informed consent. Beforehand they were informed that their decision on taking part in the study would not in any way influence their future treatment options. After their participation, the participants received CHF 40 (approx. 40 USD) reimbursement. The measures were self-report questionnaires and were assessed in one of the study languages (in written and auditory form) using a therapist-assisted computer-based screening software [59].

### Data analysis

Analyses were calculated using IBM SPSS Statistics 25.0. For each included variable, there were less than 5% of the responses missing. We did not impute any data.

As the questionnaire assessing the main outcome [58] had not been used in refugee populations so far, we ran a factor analysis to confirm that all items load on one factor. Both the Bartlett’s test of sphericity (Chi-Quadrat (15) = 245.32, *p* < 0.001) and the Kaiser–Meyer–Olkin Measure of Sampling Adequacy (KMO = 0.799) indicated that the variables are suitable for a factor analysis. Thus, we computed a principal component analysis with varimax rotation. In the analysis, there was only one factor with an eigenvalue higher than one. This finding was confirmed by the scree plot (see Additional file 1 for details). This one-factor solution explained 60% of the total variance.

Descriptive statistics were given in terms of means and standard deviations in continuous variables, and count and percentages in categorical variables. The association between demographic characteristics, exposure to potentially traumatic events, number of post-migration living difficulties, moral injury appraisals, general self-efficacy and levels of embitterment was investigated with two-sided Pearson’s correlations. All bivariate correlations were Bonferroni adjusted to correct for multiple comparisons. Traumatic experiences, post-migration living difficulties, moral injury appraisals and general self-efficacy were included as independent variables in the multiple regression analysis (method = enter). Pre-conditions for regression analyses were checked in terms of normal distribution of residuals, autocorrelation of residuals (Durbin-Watson test) and homoscedasticity were found to be satisfactory for embitterment scores. Furthermore, none of the predictors showed multicollinearity (VIF < 1.13).

## Results

### Sample characteristics

Participants had a mean age of 47.96 years (*SD* = 9.08), and the sample comprised 85.9% (*n* = 61) males from a variety of refugee backgrounds. Further sample characteristics are shown in Table 1.

**Table 1:** Sample characteristics (*N* = 71)

Sample characteristic	*M* (*SD*)/*n* (%)
Age	47.96 (9.08)
Gender (male)	61 (85.9%)
Duration of stay in CH (in yrs.)	13.87 (7.11)
*Legal status*	
Asylum seeker	1 (1.4%)
Temporary admission	5 (7.0%)
Recognized refugee	18 (25.4%)
Residency	32 (45.1%)
Citizenship	15 (21.1%)
*Marital status*	
Single	12 (16.9%)
In a relationship/married	45 (63.4%)
Widowed/divorced	14 (19.7%)
*Country of origin*	
Turkey	42 (59.2%)
Iran	6 (8.5%)
Sri Lanka	6 (8.5%)
Bosnia	3 (4.2%)
Iraq	4 (5.6%)
Other	10 (14.1%)
*Education (in yrs.)*	
Less than 4	9 (12.7%)
4–8	19 (26.8%)
8–12	16 (22.5%)
More than 12	27 (38.0%)
*Employment status*	
Full-time	9 (12.7%)
Part-time	15 (21.1%)
Unemployed	27 (38.0%)
Retired/homemaker	20 (28.2%)

Participants reported exposure to a mean of 12.76 (*SD* = 3.90) potentially traumatic events. The most frequently reported potentially traumatic events were exposure to torture (93.0%), combat situation (81.7%) and non-sexual assault by a stranger (80.3%). Despite a rather long average duration of stay in Switzerland, participants reported an average number of 7.79 (*SD* = 3.63) post-migration living difficulties. Most prominent were difficulties regarding worries about family back home (81.7%), being unable to return to home country in an emergency (73.2%) and loneliness, boredom or isolation (73.2%). The participants reported medium to high moral injury appraisals (*M* = 8.52, *SD* = 4.76) (range 0 to 15) and low to intermediate perceived self-efficacy with a mean of *M* = 23.38 (*SD* = 6.99) (range 10 to 40). Furthermore, they showed medium to high embitterment with a mean sum score of *M* = 15.83 (*SD* = 4.71) (range 0 to 24). Elevated or high embitterment scores (mean embitterment > 2.2) were reported by 67.6% (*n* = 48) of the sample.

### Correlation analysis and regression analysis

Detailed results of the correlation and regression analysis are displayed in Tables 2 and 3. Pearson correlations of embitterment with socio-demographic characteristics, traumatic experiences, post-migration living difficulties, moral injury appraisals and general self-efficacy revealed that post-migration living difficulties, moral injury appraisals and general self-efficacy were significantly associated with feelings of embitterment. We included traumatic experiences, post-migration living difficulties, moral injury appraisals and general self-efficacy as independent variables in the multiple regression analysis. The analysis revealed that moral injury appraisals, as well as general self-efficacy levels were significant predictors of embitterment (*F*(4,70) = 9.40, *p* < 0.01). The model proved to be significant and accounted for 32.4% of the variance.

**Table 2:** Pearson's correlations of embitterment with socio-demographic characteristics, traumatic experiences, post-migration living difficulties, moral injury appraisals and general self-efficacy (*N* = 71)

Measure	Gender	Stay	PTE	PMLD	MI	GSE	EMB
Gender	–	− .09	− .22	− .09	− .11	.09	− .05
Stay	− .09	–	.05	− .24	.08	− .15	.15
PTE	− .22	.05	–	.13	.20	− .10	.15
PMLD	− .09	− .24	.13	–	.07	− .09	.24
MI	− .11	.08	.20	.07	–	− .11	.43**
GSE	.09	− .15	− .10	− .09	− .11	–	− .43**
EMB	− .05	.15	.15	.24	.43**	− .43**	–

Stay*,* Duration of stay in Switzerland; PTE*,* Potentially traumatic events; PMLD*,* Post-migration Living difficulties; MI*,* Moral injury-other appraisals; GSE*,* General self-efficacy, EMB*,* Embitterment

*p < .01; ** *p* < .002 (*p*-values are Bonferroni adjusted for five related constructs)

**Table 3:** Multiple hierarchical regression analysis (*N* = 71)

DV	IV	B	SE B	β	t	F	R^2^	Adj. R^2^
Embitterment						9.40**	.36	.32
	PTE	0.03	0.12	.02	0.21			
	PMLD	0.22	0.13	.17	1.74			
	MI	0.37	0.10	.37**	3.66			
	GSE	− 0.25	0.07	− .37**	-3.76			

DV, Dependent variable; IV, Independent variable; Stay, duration of stay in Switzerland; PTE, Potentially traumatic events; PMLD, Post-migration living difficulties; MI, Moral injury-other appraisals; GSE, General self-efficacy

*p < .05; **p < .01

## Discussion

This exploratory cross-sectional study examined the role of embitterment and its association with trauma exposure, post-migration living difficulties, moral injury, and self-efficacy in a clinical sample of traumatized refugees and asylum seekers. Elevated levels of embitterment were reported frequently, with more than 65% of our sample scoring above the cut-off. Multiple regression analysis demonstrated that moral injury appraisals and general self-efficacy, but neither trauma exposure nor the number of PMLDs significantly predicted feelings of embitterment.

Compared to clinical non-refugee samples [41], we found a substantially higher rate of elevated embitterment in our sample. This finding might be explained by the fact that refugees frequently experience injustice, offensive treatment and humiliation [4] and might therefore be at a higher risk of developing feelings of embitterment compared to a clinical sample from Western countries. A study by Linden and Teherani [47] points in the same direction. They compared Iranian refugees with Iranian non-refugee emigrants, and found significant group differences indicating that the refugee experience (including pre—and post-migration adversities) is linked to higher embitterment levels [47]. Alternatively, transcultural aspects regarding the understanding of suffering and fate might explain differing findings in Western and non-Western samples [60]. For instance, studies comparing cultural concepts of disease between Turkish and German inpatient samples have found that Turkish immigrants often reported an external illness-related locus of control and frequently believed that their illness was related to fate or a religious cause [61, 62]. In his theoretical model on embitterment, Znoj [30] highlights that embitterment is possibly related to an external locus of control, indicating that in our study, the development of high embitterment might not only be related to the refugees’ experiences but also to cultural influences. However, the influence of culture on an external illness-related locus of control and its link to embitterment has not been examined in this study and should be addressed by future research.

The association between higher moral injury appraisals and higher levels of embitterment is in line with earlier conceptualisations on the aetiology of embitterment [28, 30]. The concept of moral injury has been developed based on research with veterans [44] but has recently been applied to the refugee context as well [19, 45, 46], as it might be one of the psychological mechanisms contributing to the development of mental health problems following displacement. A study investigating moral injury appraisals in refugees revealed that moral injury explained a substantial amount of variance in posttraumatic stress disorder (16%), depression (16%), explosive anger (10%) and mental health-related quality of life (10%) [19]. The association between moral injury appraisals and elevated embitterment levels in our study suggests that the subjective interpretation (e.g. perceived injustice) of the refugees’ experiences throughout the whole trajectory plays a major role in the development of mental health symptoms (analogous to cognitive models on PTSD, see Ehlers and Clark [63]) [19, 31].

Our data revealed a negative association between embitterment and self-efficacy. In previous research, the belief in one’s capacity to achieve a desired goal has emerged as an important factor of psychological well-being. Self-efficacy, or the lack of it, has been related to anxiety and depression [64], PTSD and posttraumatic growth [33, 65, 66], and health related quality of life [67, 68]. Even though quite a few studies have analyzed the relation between self-efficacy and mental health in refugees and asylum seekers [21, 69, 70], no other research has, to our knowledge, examined the relation between embitterment and self-efficacy in this population before. Znoj’s [30] theoretical model specified an external locus of control, as well as hopelessness as constituents of embitterment. The model does not include self-efficacy, however self-efficacy, locus of control and hope are similarly defined, as they all incorporate a possibility for change and a positive orientation towards the future [71–73]. Thus, the diametrical association between embitterment and self-efficacy was somewhat expected and might add some additional explaining variance to Znoj’s [30] model of embitterment. However, these findings are preliminary and of exploratory nature, and therefore, more comprehensive investigation of the relationship between these constructs and their association with embitterment is needed.

Surprisingly, trauma exposure was not significantly associated with embitterment. This is remarkable since research has shown that experiences related to war and violence may foster moral transgressions (e.g. 45) and low levels of self-efficacy [66], which, in turn, may favor the development of embitterment. However, we solely assessed the total count of trauma categories experienced, but neither the frequency nor the effect of specific subtypes (e.g. interpersonal trauma). A similar pattern emerged between post-migration living difficulties and embitterment. This result is somewhat unexpected, considering the continuing adversities refugees frequently face in their host countries [26, 74]—which is the opposite of what they had expected when they fled their country of origin. As with trauma exposure, it is possible that the sheer quantitative exposure to PMLD, as assessed in this study, is not relevant with regard to the emergence of embitterment, while the qualitative exposure to particular post-migration stressors, for instance in terms of rejected asylum claims, labour market restrictions, or discrimination may be. This interpretation might be supported by a similar (non-) correlation between trauma exposure, PMLD and moral injury appraisals—even though moral injury must be assumed to be the consequence of some of these experiences. Thus, the relation between trauma exposure, PMLD and embitterment remains unclear and should be addressed in future work with larger sample sizes, preferably using longitudinal study designs with multiple assessments.

### Implications

The level of embitterment in our sample was substantially higher than in clinical non-refugee samples [41]. This finding suggests, if replicated, that embitterment might be a relevant aspect regarding refugee mental health. Individuals reporting high levels of embitterment also frequently suffer from other mental health problems [42, 75]. Therefore, diagnostic screening for embitterment might be considered when treating mentally ill refugees and asylum seekers.

Second, embitterment was highly present in our sample even though all participants were in treatment. This suggests that standard psychotherapeutic interventions applied when working with refugees may not adequately address embitterment. In addition, previous research has shown that embitterment might hamper the recovery process [76]. Alternative approaches such as “wisdom therapy” [77] or interventions focusing on forgiveness [78, 79] have been shown to have promising effects and might add some additional benefit when treating embitterment. Although these interventions were mostly applied in Western patients, clinicians might find such interventions helpful when working with refugees and asylum seekers. Furthermore, interventions targeting moral injury appraisals and enhancing self-efficacy may add some additional therapeutic value, as these two concepts were associated with high levels of embitterment.

A third implication relates to the diametrical association between general self-efficacy and embitterment. By nature of their displacement, but also after resettlement, refugees often find themselves in unswayable environments with very little control over their lives. On top, they are frequently confronted with restrictive migration policies, e.g. with regard to access to labour market, family reunion, or accommodation [80–82]. Living with so many restrictions without experiencing empowerment or mastery can add to a diminished belief in the ability to achieve desired outcomes and the capacity to exert control [82]. Low levels of self-efficacy might then, in turn, contribute to poor mental health outcomes, e.g., feelings of embitterment, and impede psychosocial integration even when opportunities arise [10]. Policy makers should be aware that the downside of such restrictions usually implemented in order to avoid pull factors might include negative effects on mental health of refugees and on their capacity with regard to psychosocial integration.

Therefore, a recent study by Tip and colleagues [82] highlights the need for migration policies adapted to increase the belief in one’s abilities and maximize self-determination of refugees, e.g. by enabling early language acquisition and timely access to the labour market.

### Limitations

A major limitation is the rather small sample size, which raises questions about the representativity of the sample. The majority of the participants came from Turkey and had a rather long average duration of stay in Switzerland and a secure legal status. Therefore, the results might not be comparable to other refugee populations, e.g. other ethnicities or individuals awaiting the decision on their asylum claim. Moreover, the distribution of men and women in the sample was uneven, with 85% of the participants being male. Additionally, all participants underwent treatment and had been in different stages of therapy at the time of the assessment. The length of treatment was not assessed and thus, the effect of treatment on our findings remains unclear.

A second limitation is the cross-sectional study design, which does not allow for causal inferences.

A third limitation is the use of the short form of the Bern Embitterment Inventory [58], a version which has not yet been published and validated. Moreover, the research on embitterment in different cultures is still in its infancy and thus, neither the original scale of the BEI [57] nor the short version [58] have been used transculturally yet. In addition, the Bern Embitterment Inventory is a self-report questionnaire that measures the broad construct of embitterment rather than reactive or posttraumatic embitterment. Linden and colleagues [40] found rather high correlations between two questionnaires measuring embitterment, which suggests good convergent validity, yet future researchers should consider using diagnostic interviews to assess chronic (reactive) embitterment. Although a preliminary cut-off distinguishing between non-pathological embitterment and high or chronic levels of embitterment has been proposed by Znoj [57] for the original scale, future work should investigate whether this cut-off applies to the short version of the scale, as well as to different samples and populations.

## Conclusions

This exploratory study provides first insights into embitterment and associated factors in a clinical sample of refugees and asylum seekers. The number of individuals scoring above the cut-off for elevated embitterment in our sample, compared with non-clinical as well as non-refugee samples, suggests that embitterment might be of particular relevance in this population. The relationship of embitterment with moral injury, and low self-efficacy, but not with trauma exposure or PMLD, suggests that the subjective interpretation of these experiences rather than the sheer sum of adversities might play a major etiological role, which should be further examined in future longitudinal studies. In view of the high prevalence of embitterment in our sample, this condition may require more attention regarding diagnostics and alternative therapeutic procedures in refugees and asylum seekers.

## Supplementary Information

**Additional file 1**. Scree plot.

## Data Availability

The datasets generated for this study are available on reasonable request from the corresponding author.

## Abbreviations

PTE: Potentially traumatic events
PTSD: Posttraumatic stress disorder
PMLD: Post-migration living difficulties
CH: Switzerland
Yrs.: Years
DV: Dependent variable
IV: Independent variable
MI: Moral injury
GSE: General self efficacy
EMB: Embitterment
