# Supplementary figures and images for: An exploratory study of embitterment in traumatized refugees
Source: BMC Psychol. 2021 Jun 10;9:96. doi: 10.1186/s40359-021-00599-2 (PMC8193876; doi:10.1186/s40359-021-00599-2)

**Scree Plot**

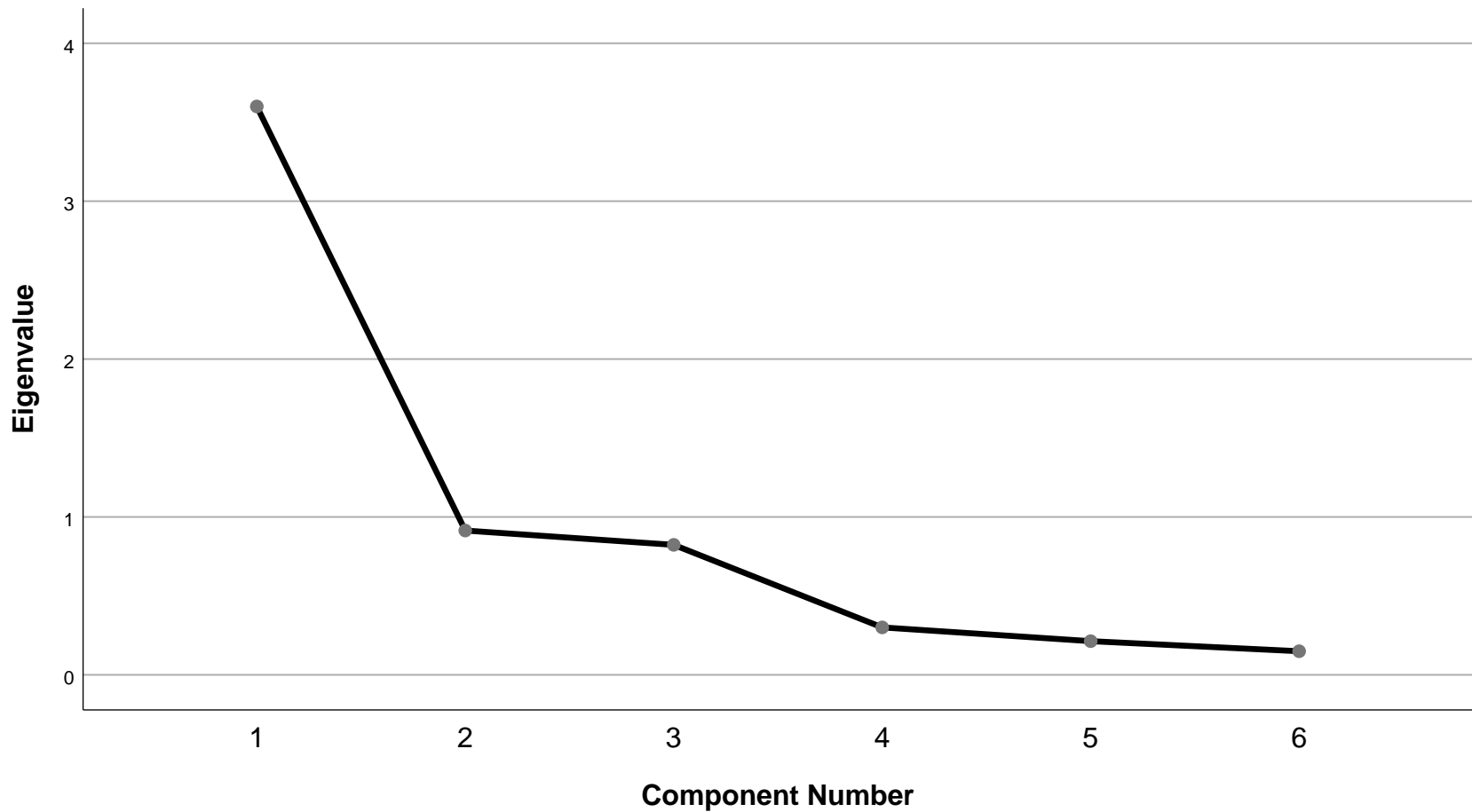

Supplement: Supplementary file 1 — Additional file 1. Scree plot. [file 40359_2021_599_MOESM1_ESM.pdf]
